# Childhood disability, social class and social mobility: A neglected relationship

**DOI:** 10.1111/1468-4446.12974

**Published:** 2022-09-05

**Authors:** Stella Chatzitheochari, Sanne Velthuis, Roxanne Connelly

**Affiliations:** ^1^ University of Warwick Coventry UK; ^2^ University of Newcastle Newcastle Upon Tyne UK; ^3^ University of Edinburgh Edinburgh UK

**Keywords:** childhood disability, intersectionality, social class, social mobility, social stratification

## Abstract

Disability theorists have long highlighted the role of institutional, social, and environmental barriers in constructing disability, emphasizing its parallels with other socially constructed axes of stratification. However, despite theoretical developments toward sociological understandings of disability, social stratification and life‐course research have largely neglected childhood disability as a social division. As a result, we still know little surrounding the socio‐economic attainment of disabled children and young people. Drawing on Next Steps data, this research note highlights stark overlooked inequalities between disabled and non‐disabled young people's activity status and social mobility in early adulthood. We specifically focus on the importance of social class for disabled young people's outcomes, emphasizing the need for intersectional analyses of disability inequalities. We also outline longitudinal survey data enhancements necessary for life‐course research on childhood disability and its intersections.

## 1 CHILDHOOD DISABILITY AND SOCIAL STRATIFICATION RESEARCH

Recent decades have witnessed an increase in the prevalence of childhood disability, a term used to refer to a wide range of mind‐body characteristics that fall outside societal expectations of learning and development (Holt, 2004). There is ample evidence surrounding the association of childhood disability with socio‐economic disadvantage: Disabled children and young people are more likely to come from disadvantaged backgrounds (Parsons & Platt, 2013; Spencer et al., 2015) and fall behind non‐disabled peers in educational, occupational, and social outcomes in adulthood (Chatzitheochari & Platt, 2019; Janus, 2009; Office for National Statistics, 2021).

Disability studies have been pivotal in highlighting the contingent and socially constructed nature of disability, drawing attention to the arbitrariness of disability classification systems and their reliance on political ideologies of “normalcy” (Davis, 1995; Powell, 2003), and to macro‐ and micro‐level processes that contribute to segregation and othering of people with impairments and long‐term conditions (Barnes & Oliver, 1993). These theoretical developments have led to a growth of empirical research on disability, which has mostly focused on adult populations, neglecting children and young people (Watson, 2012). At present, sociological research on disabled children and young people remains concentrated in niche fields of education studies, focusing on schooling and learning, including processes of stigmatization in school settings (Armstrong et al., 2011). In contrast, childhood disability remains a largely neglected social division within mainstream empirical sociology, despite longstanding calls for social stratification and life‐course research on disability‐related disadvantage (Jenkins, 1991; Powell, 2003).

An important shortcoming of existing scholarship is the framing of childhood disability as a monolithic ascriptive status, with little consideration of its intersections with other social divisions. Although scholars have emphasized the importance of intersectional understandings of hierarchies of disadvantage among heterogeneous disabled populations (Jenkins, 1991; Shifrer & Frederick, 2019), there is a lack of large‐scale longitudinal datasets that provide sufficient numbers of disabled children and/or young people for intersectional analyses in the UK. As a result, we know little surrounding classed disparities in socio‐economic outcomes of disabled children and young people. This is a glaring omission given the centrality of social class for social reproduction processes (Bourdieu & Passeron, 1990).

This note aims to encourage social stratification research on childhood disability by drawing attention to its intersections with social class with respect to social mobility in early adulthood. To our knowledge, ours is the first examination of this relationship, which has been consistently neglected within mainstream sociology. We present visualizations of stark outcome disparities by parental social class, highlighting the need for intersectional understandings of disability‐related disadvantage.

## 2 NEXT STEPS DATA

We graphically display classed differences in social mobility by disability status, drawing on data from Next Steps, a nationally representative cohort study of approximately 16,000 individuals born in 1989–1990 in England (University College London, Institute of Education and Center for Longitudinal Studies, 2021). Next Steps is the most recent cohort study that can be used to examine contemporary patterns of social mobility and transitions to adulthood of disabled adolescents in England.

Social class is measured with the 3‐class version of the National Statistics Socioeconomic Classification (NS‐SEC), the official socio‐economic classification in the UK, which operationalizes class based on employment relations and occupational conditions (Rose & Pevalin, 2003). For parental social class, we consider the highest occupational position reported by either parent in the first wave of data collection, when cohort members were 13/14 years old. Young people's socio‐economic position is measured at age 25. NS‐SEC is based on individuals' occupation and employment status (whether they are employed, self‐employed, or an employer) and it is thus only available for those in employment. The “not in employment” category includes those who were unemployed, in education, sick or disabled, and looking after family at age 25. We acknowledge that young people have not reached occupational maturity at age 25, and that transitions of disabled youth may take longer to be completed than those of non‐disabled peers (Janus, 2009). Data limitations currently preclude the analysis of outcomes at a later age. However, we contend that inspecting outcomes at this age provides initial insights into the ongoing processes of social reproduction for this group.

Childhood disability status was measured at the same age as parental social class. Those identified as disabled had a long‐standing illness, condition, or impairment affecting school attendance and/or the ability to complete homework, and/or a special educational need of any kind.^1^ These measures align with the understanding of disability in the UK Equality Act 2010 which is rooted in the medical model and locates disability within individual conditions and illnesses, disregarding the role of societal barriers for processes of disablement.

Results are adjusted for non‐response and attrition using wave‐specific weights provided in Next Steps.

## 3 CHILDHOOD DISABILITY, SOCIAL CLASS, AND SOCIAL MOBILITY IN ENGLAND

**FIGURE 1:**
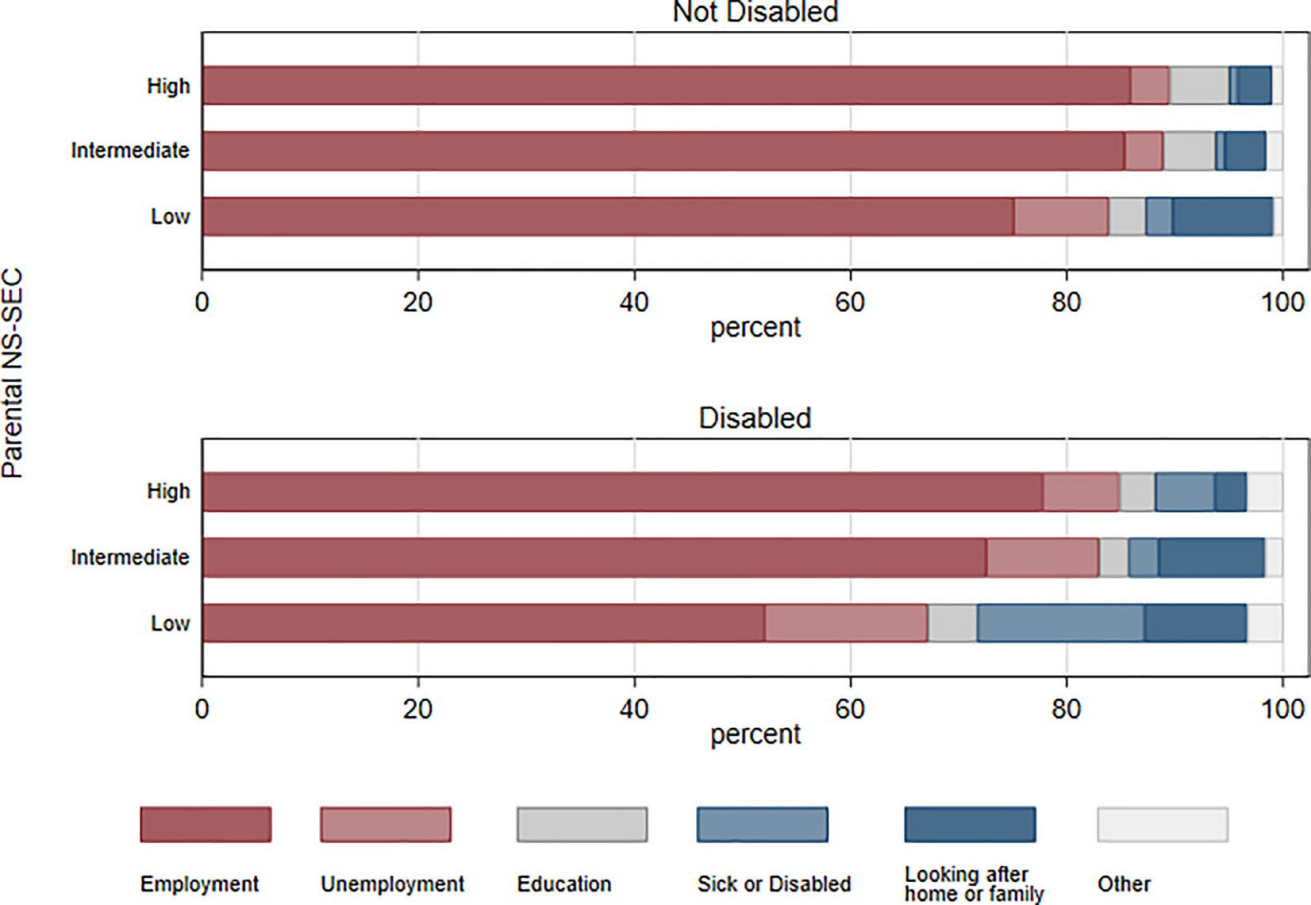
Main activity at age 25, by disability status and parental NS‐SEC. *Source*: Next Steps, Wave 1–8. *Notes*: *n* = 6004 (not disabled); *n* = 849 (disabled). Weights applied. Low NS‐SEC includes (1) lower supervisory and technical occupations, (2) semi‐routine occupations, (3) routine occupations. Intermediate NS‐SEC includes (1) intermediate occupations, (2) small employers and own account workers. High NS‐SEC includes (1) higher managerial, professional, and administrative occupations, (2) lower managerial, professional, and administrative occupations

Figure 1 displays main activities reported by cohort members at age 25, revealing pronounced inequalities by disability status and social class (see Table A1 for further details). Although the majority of cohort members have entered the labor market by age 25, disabled young people have substantially lower participation rates compared to non‐disabled peers. Disability disparities vary substantially by parental social class position: There is a 23‐percentage point differential in the probability of employment between disabled and non‐disabled young people from the most disadvantaged social class origin, as opposed to an 8‐percentage point differential between those from the most advantaged social class origin. There are similar classed differences in reports of unemployment and sickness/disability as main activities (Figure 1). These patterns indicate that disability has a disproportionate effect on young people from disadvantaged social class backgrounds with respect to early adulthood economic activity, and thus warrant further investigation.

Figure 2 demonstrates stark disability inequalities in social class origins and destinations (see Tables A2 and A3 for further details). Disabled young people are more likely to come from the most disadvantaged social class origin (43% as opposed to 32% for non‐disabled peers). The thickness of the flow lines in Figure 2 demonstrates that non‐disabled young people are almost twice as likely to experience upward mobility compared to non‐disabled counterparts (23% as opposed to 13%). Furthermore, class reproduction is far less common for this group: For example, only 10% of disabled youth have parents from the most advantaged social class origin and remain in similar class positions at age 25, as opposed to 21% of non‐disabled young people, for whom class stability is the most common outcome. The proportion of those not in employment also varies substantially by disability status, with disabled young people from the most disadvantaged backgrounds disproportionately more likely to fall into this category (21%). These findings cannot be attributed to disability status differences in the internal composition of 3 broad parental social class groups (see Table A4).

FIGURE 2Social class origins and destinations at age 25, by disability status. *Source*: Next Steps, Wave 1–8. *Notes*: *n* = 6004 (not disabled); *n* = 849 (disabled). Weights applied. Low NS‐SEC includes (1) lower supervisory and technical occupations, (2) semi‐routine occupations, (3) routine occupations. Intermediate NS‐SEC includes (1) intermediate occupations, (2) small employers and own account workers. High NS‐SEC includes (1) higher managerial, professional, and administrative occupations, (2) lower managerial, professional, and administrative occupations. Not in employment category includes participants who are in full‐time education, unemployed, sick or disabled, and looking after family. Thickness of the lines represent people moving from origin to destination social classes. Numbers under the origins and destinations labels indicate the weighted proportion in each group. For example, in the bottom left of the graph for disabled young people, 43% indicates the percent of disabled young people whose parents were in low NS‐SEC occupations. Similarly, the 35% at the top of the same graph, indicates the percent of young people who are not in employment at age 25. The numbers within the flows indicate the percentages within each flow/trajectory. For example, the 11% on the right near the bottom of the graph for disabled young people indicates the percent whose parents were in high NS‐SEC occupations who are in low NS‐SEC occupations at age 25, thus experiencing downward mobility

**Figure:**
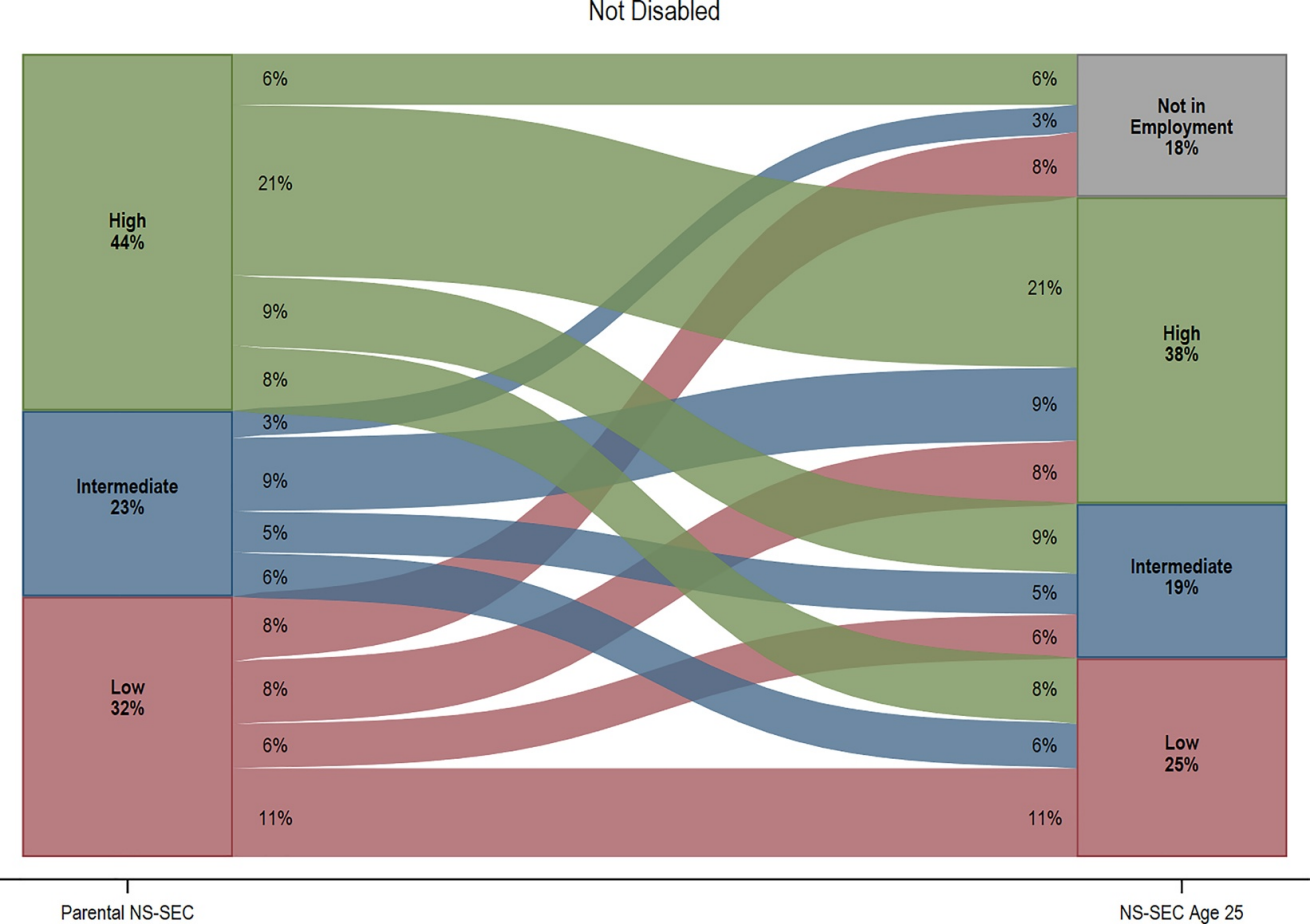

**Figure:**
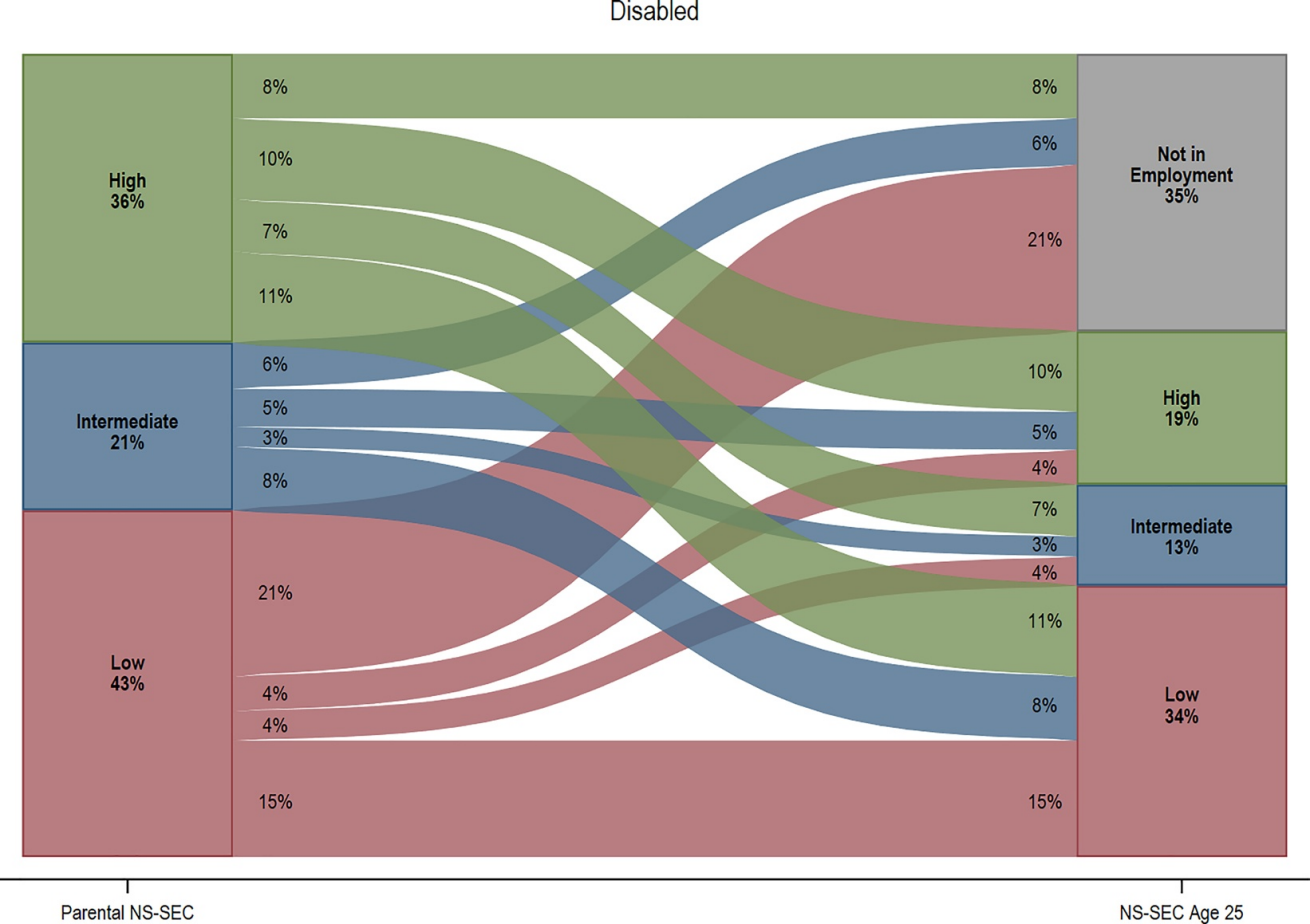

## 4 LOOKING AHEAD: AVENUES FOR LONGITUDINAL SURVEY DATA COLLECTION

This note sought to encourage social stratification research on childhood disability, with a particular emphasis on its intersections with social class background. We demonstrated radical differences in the social mobility chances of disabled and non‐disabled young people in England. Disabled young people are notably less likely to experience upward mobility and more likely to experience downward mobility and/or to be out of employment at age 25, compared to their non‐disabled counterparts. Existing theoretical and empirical literature suggests several pathways through which these inequalities may come into play, including differences in educational attainment, structural discrimination, stigmatization, as well as impairment‐specific barriers and considerations (Chatzitheochari & Platt, 2019; Janus, 2009; Jenkins, 1991; Link & Phelan, 2001; Powell, 2003). However, there is a general lack of longitudinal research that sheds light into the different mechanisms implicated in the reproduction of disability‐related disadvantage among young people. In this final section, we briefly discuss existing barriers for research focusing on this analytical endeavor in the UK context, and we briefly outline future avenues that can enable social stratification analyses of childhood disability and its intersections.

Cohort studies like Next Steps are crucial for the generalized purpose of highlighting trajectories of disadvantage by disability status and variation by key contextual factors (Altman, 2014). Despite the wealth of social science data resources available to UK researchers, there are notable limitations in data availability for the study of childhood disability: The Millennium Cohort Study will provide opportunities to examine disability‐related disadvantage as the cohort matures. Unfortunately, there will then be a gap of more than 20 years before any new potential birth cohort data become available. Valuable youth datasets such as the Youth Cohort Study of England and Wales are now defunct. Multipurpose household panel studies such as *Understanding Society* provide very small sample sizes to examine school year cohorts. Administrative datasets arguably hold limited promise for sociological analyses of youth transitions due to lack of appropriate variables, and intransient difficulties in accessing and linking administrative data resources in the UK context (Connelly et al., 2016).

Existing studies provide information on official school recognition and Special Educational Needs (SEN) support, allowing researchers to address important debates surrounding provision efficacy and/or institutional scarring. However, they rarely allow analyses by type of disability/special educational need, due to small sample sizes, which are further reduced by sample attrition (Chatzitheochari & Platt, 2019). This constitutes an important barrier for understanding the formation of inequalities documented in our note: There are impairment‐specific limitations that prevent access to certain types of occupations, while different disabilities are also subject to varying structural and cultural “ableisms” and stigmas (Campbell, 2009). This means that there may be different causal paths leading to similar outcomes of individuals with different disabilities. Similarly, classed disparities in socio‐economic outcomes may be driven by class differences in types of impairments/conditions experienced by young people. Oversampling of people with different impairments/conditions can allow researchers to answer such questions of intra‐ and inter‐categorical nature in the future.

A central question is the extent to which intersectional inequalities in educational attainment account for the social mobility patterns presented in this note. Unfortunately, the majority of longitudinal studies in the UK provide low quality vocational qualifications data, which are particularly common for this population. Enhanced interview questionnaires and/or data linkage with Individualized Learner Records (ILR) are necessary for investigations of the role of educational careers and attainment in trajectories of downward mobility of disabled young people. Such data enhancements are also necessary for much needed cross‐national analyses of disabled children and young people's socio‐economic outcomes and life chances.

Improvements in other measures is also crucial: Questions on experiences of discrimination in school settings and/or the workplace, which are occasionally employed for other identities like gender and ethnicity, could also be used for disability. Similarly, although stigma is particularly difficult to capture in a social survey context (Van Brakel, 2006), a wide range of psychosocial variables can provide indirect measures of this concept, which occupies a central place in sociological explanations of disability‐related disadvantage (Link & Phelan, 2001).

To conclude, while we acknowledge that other methodological approaches are necessary to fully understand the formation of the inequalities documented in this note, we argue that enhanced longitudinal resources will offer novel analytical opportunities for social stratification research, ultimately contributing to the inclusion of childhood disability in mainstream accounts of social inequality.

## CONFLICTS OF INTEREST

The authors declare that there is no conflict of interest that could be perceived as prejudicing the impartiality of the research reported.

## Supporting information

Supporting Information S1

## Data Availability

The data that support the findings of this study are openly available in UK Data Service at http://doi.org/10.5255/UKDA‐SN‐5545‐8 reference number SN: 5545.

## ENDNOTE

^1^
We used Wave 2 information for 4% of young people with missing information on disability status in Wave 1. Young people whose parents only reported “English as second language” as their special educational need were not classified as disabled in our analysis. Readers are referred to Velthuis and Chatzitheochari (2021) for further details on these measures.
