# Supplementary material for: Childhood disability, social class and social mobility: A neglected relationship
Source: Br J Sociol. 2022 Sep 5;73(5):959–66. doi: 10.1111/1468-4446.12974 (PMC10087124; doi:10.1111/1468-4446.12974)
Supplement: Supplementary file 1 — Supporting Information S1 [file BJOS-73-959-s001.docx]

**Appendix**

| **Table A1*:****Disability differentials in main activities at age 25, by parental NS-SEC at age 13/14* | | | | | | | | | | | |  | |  | | |
| --- | --- | --- | --- | --- | --- | --- | --- | --- | --- | --- | --- | --- | --- | --- | --- | --- |
|  | *High NS-SEC (percentage in each main activity)* | | | | | *Intermediate NS-SEC (percentage in each main activity)* | | | | *Low NS-SEC (percentage in each main activity)* | | | | | | |
|  | | Disabled  (n = 363) | Not disabled  (n = 2,859) | Dif. | Disabled  (n = 182) | | Not disabled (n = 1,388) | Dif. | Disabled  (n = 304) | | Not disabled (n = 1,757) | | Dif. | |  |  |
| Employment | | 77.8 | 85.8 | -8 | 72.5 | | 85.3 | -12.8 | 51.9 | | | 75 | | -23.1 | |  |
| Unemployment | | 7.2 | 3.7 | 3.5 | 10.7 | | 3.6 | 7.1 | 15.1 | | | 8.9 | | 6.2 | |  |
| Education | | 3.3 | 5.5 | -2.2 | 2.6 | | 4.8 | -2.2 | 4.6 | | | 3.4 | | 1.2 | |  |
| Sick or disabled | | 5.6 | 0.8 | 4.8 | 2.9 | | 0.9 | 2 | 15.6 | | | 2.5 | | 13.1 | |  |
| Looking after home or family | | 2.9 | 3.2 | -0.3 | 9.7 | | 3.8 | 5.9 | 9.4 | | | 9.3 | | 0.1 | |  |
| Other | | 3.2 | 1.0 | 2.2 | 1.6 | | 1.6 | 0 | 3.4 | | | 0.9 | | 2.5 | |  |
| Total | | 100 | 100 |  | 100 | | 100 |  | 100 | | | 100 | |  | |  |
| *Source:* Next Steps, Wave 1-8.  Notes: n =6,853. Unweighted *Ns*; weighted percentages. | | | | | | | | | | | | | |  | | |

| **Table A2.** *Social mobility at age 25, by disability status* | | | | |  |  |  |
| --- | --- | --- | --- | --- | --- | --- | --- |
|  |  |  |  | **NS-SEC at age 25** |  |  |  |
|  |  |  |  | *Disabled* |  |  |  |
| **Parental NS-SEC at age 13/14** | | | High | Intermediate | Low | Not in employment | Total |
| High (n=363) | |  | 28.0 | 18.1 | 31.8 | 22.2 | 100 |
| Intermediate (n=182) | |  | 22.7 | 12.0 | 37.8 | 27.5 | 100 |
| Low (n=304) | |  | 9.9 | 8.3 | 33.7 | 48.1 | 100 |
| Total (n=849) | |  | 19.0 | 12.6 | 33.9 | 34.5 | 100 |
|  |  |  |  |  |  |  |  |
|  |  |  |  | *Not Disabled* |  |  |  |
| **Parental NS-SEC at age 13/14** |  |  | High | Intermediate | Low | Not in employment | Total |
| High (n=2859) | |  | 47.8 | 19.6 | 18.4 | 14.2 | 100 |
| Intermediate (n=1,388) | |  | 39.3 | 21.9 | 24.1 | 14.7 | 100 |
| Low (n=1,757) | |  | 24.1 | 16.9 | 34.0 | 25.0 | 100 |
| Total (n=6,004) | |  | 38.1 | 19.3 | 24.8 | 17.8 | 100 |

Source: Next Steps, Wave 1-8.

*Notes*: n=6,853. Unweighted *Ns*; weighted percentages.

| **Table A3: Patterns of social mobility at age 25, by disability status** | | | | | | | |
| --- | --- | --- | --- | --- | --- | --- | --- |
|  | Disabled  (n = 849) | | Not disabled (n = 6,004) | | All  (n = 6,853) | |  |
| Upward mobility | 12.6 | | 22.4 | | 20.7 | |  |
| Stability | 27.1 | | 37.3 | | 35.6 | |  |
| Downward mobility | 25.8 | | 22.4 | | 23.0 | |  |
| Not in employment | 34.5 | | 17.8 | | 20.7 | |  |
| Total | 100 | | 100 | | 100 | |  |
| Source: Next Steps, wave 1-8.  *Notes*: n=6,853. Unweighted *Ns*; weighted percentages. | | | | | | |  |
| **Table A4:** *Composition of broad parental NS-SEC classes at age 25, by disability status* | | | | | | | |
| *High parental NS-SEC* | | Disabled (n = 363) | | Not disabled  (n = 2,859) | | All  (n = 3,222) | |
| Higher managerial, professional, and administrative occupations | | 26.7 | | 32.3 | | 31.6 | |
| Lower managerial, professional, and administrative occupations | | 72.4 | | 67.7 | | 68.4 | |
| Total | | 100 | | 100 | | 100 | |
|  | |  | |  | |  | |
| *Intermediate parental NS-SEC* | | Disabled (n = 182) | | Not disabled  (n = 1,388) | | All  (n = 1,570) | |
| Intermediate occupations | | 51.9 | | 54.2 | | 53.9 | |
| Small employers and own account workers | | 48.1 | | 45.8 | | 46.1 | |
| Total | | 100 | | 100 | | 100 | |
|  | |  | |  | |  | |
| *Low parental NS-SEC* | | Disabled (n = 304) | | Not disabled  (n = 1,757) | | All  (n = 2,061) | |
| Lower supervisory and technical occupations | | 27.8 | | 30.3 | | 29.8 | |
| Semi-routine occupations | | 38.7 | | 43.6 | | 42.6 | |
| Routine occupations | | 33.5 | | 26.1 | | 27.7 | |
| Total | | 100 | | 100 | | 100 | |
| Source: Next Steps, Waves 1-8.  *Notes:* n = 6,853. Unweighted *Ns*; weighted percentages. | | | | | | | |
